# Change in the prevalence of metabolic syndrome in a population of medical students: 6-year follow-up

**DOI:** 10.1186/s40200-015-0216-4

**Published:** 2015-11-25

**Authors:** Fernando J. Lavalle, Jesús Z. Villarreal, Juan Montes, Leonardo G. Mancillas, Susana E. Rodríguez, Patricio González, Reynaldo Lara

**Affiliations:** Departamento de Medicina Interna, Hospital Universitario Dr. José Eleuterio, Universidad Autónoma de Nuevo León, Servicio de Endocrinología y, González, Av. Francisco I. Madero y Av. Gonzalitos s/n, Colonia Mitras Centro, Monterrey, Nuevo León C.P. 64460 Mexico

**Keywords:** Metabolic syndrome, Mexican population, Students

## Abstract

Students of a university hospital were assessed in 2007 and later in 2013 to determine the prevalence of metabolic syndrome. Statistical analysis was done with SPSS version 17.0.

A total of 213 students were evaluated in both 2007 and 2013 (48.3 % women and 51.7 % men). The diagnosis of overweight and obesity increased from 24.9 to 37.1 % (*p* < 0.05), central obesity from 17.8 to 28.6 % (*p* < 0.05), and prevalence of metabolic syndrome from 9.8 to 14.5 % (*p* ≥ 0.05); up to 20 % in male gender. It is important to implement programs for early diagnosis of metabolic syndrome.

Metabolic syndrome is a group of vascular and metabolic risk factors, including insulin resistance, dyslipidemia and hypertension [1–4]. Cardiovascular disease is its primary clinical outcome and it is estimated that people with this condition have a 3-fold higher risk for an acute coronary syndrome or a cerebral vascular event; also the risk of mortality is 2 times higher [5]. Other diseases related to metabolic syndrome are type 2 diabetes mellitus, polycystic ovary syndrome, fatty liver, asthma, sleep disorders, and cancer [6].

Some studies show that young adults who enter school have poor health habits (lack of physical activity, tobacco and alcohol use, and unhealthy diet). Additionally, it has been noted that first-year students gain weight faster than the average adult [7–10]. Morrison et al. noted that the diagnosis of metabolic syndrome in infancy, predicts cardiovascular disease 25 years later [11]. The prevalence of metabolic syndrome in students according to the literature varies from 1.3 to 14.3 % [12–16].

The aim of this study is to evaluate the change in the prevalence of metabolic syndrome in a population of medical students over a six-year period using the definition of the International Diabetes Federation.

## Research design and methods

We performed a prospective, descriptive and non-randomized study in freshmen medical students from the Faculty of Medicine registered in August 2007 (under 20 years of age) and that remained registered in August 2013 (under 30 years of age), in a University Hospital. Pregnant subjects were excluded. The study design was approved by the institution’s ethics committee and informed consent was obtained from all the participants. On enrollment day, students who accepted to participate in the study, anthropometric measurements were taken, blood samples to perform blood chemistry and lipid profile were drawn, and blood pressure was measured.

To determine metabolic syndrome, International Diabetes Federation criteria were used. Abdominal obesity was defined as a waist circumference ≥ 90 cm in men and ≥ 80 cm in women, plus two of the following: fasting plasma glucose greater than 100 mg/dL or a prior diagnosis of DM2, triglycerides ≥ 150 mg/dL, HDL cholesterol ≤ 40 mg/dL in men or ≤ 50 mg/dL in women, and a systolic blood pressure ≥ 130 mmHg or a diastolic blood pressure ≥ 85 mmHg.

Economic status, hometown and parents job were not evaluated in this study population.

## Results

Two thousand thirteen students were included in the study: 48.3 % women and 51.7 % men. Demographic and laboratory characteristics of the population are shown in Tables 1, 2 and 3.

**Table 1:** Anthropometric, clinical and biochemical characteristics of the total population

Variable	Total population (*n* = 213)		*p*
	2007	2013	
Age (years)	17.27 (1.11)	23.26 (1.09)	<0.05
Weight (kg)	63.71 (13.93)	68.41 (14.57)	<0.05
BMI (kg/m^2^)	22.98 (4.09)	24.37 (4.04)	<0.05
Waist (cm)	76.27 (11.02)	80.75 (10.79)	<0.05
Systolic BP (mm Hg)	115.51 (11.70)	119.61 (15.18)	<0.05
Diastolic BP (mm Hg)	73.11 (8.08)	73.44 (10.10)	≥0.05
Glucose (mg/dL)	87.69 (24.37)	84.86 (12.66)	≥0.05
Triglycerides (mg/dL)	146.93 (77.13)	81.85 (43.81)	<0.05
HDL Cholesterol (mg/dL)	42.97 (12.27)	40.23 (12.37)	<0.05
LDL Cholesterol (mg/dL)	114.30 (56.01)	95.71 (34.81)	<0.05
Total Cholesterol (mg/dL)	184.95 (61.71)	151.73 (35.38)	<0.05

Data are shown as Mean ± standard deviation

*BMI* body mass index; *BP* blood pressure; *LDL* low-density lipoprotein cholesterol; *HDL* high-density lipoprotein cholesterol. Student’s *t* test was used for the analysis with a *p* <0.05 being considered statistically significant

**Table 2:** Anthropometric, clinical and biochemistry characteristics of the female population

Variable	Women (*n* = 103)		*p*
	2007	2013	
Age (years)	17.02 (1.03)	23.1 (1.04)	<0.05
Wight (kg)	56.43 (10.66)	59.98 (11.65)	<0.05
BMI (kg/m^2^)	22.42 (3.90)	23.58 (4.23)	<0.05
Waist (cm)	71.11 (9.19)	75.08 (9.62)	<0.05
Systolic BP (mm Hg)	114.3 (11.69)	110.6 (11.3)	≥0.05
Diastolic BP (mm Hg)	72.09 (7.55)	70.35 (9.07)	<0.05
Glucose (mg/dL)	87.93 (24.85)	83.45 (12.70)	≥0.05
Triglycerides (mg/dL)	132.34 (60.21)	70.12 (24.13)	<0.05
HDL Cholesterol (mg/dL)	44.27 (11.93)	43.29 (13.09)	≥0.05
LDL Cholesterol (mg/dL)	122.07 (58.89)	94.44 (37.33)	<0.05
Total Cholesterol (mg/dL)	190.98 (67.70)	150.88 (38.73)	<0.05

Data are shown as mean ± standard deviation

*BMI* body mass index; *BP* blood pressure; *LDL* low-density lipoprotein cholesterol; *HDL* high-density lipoprotein cholesterol. Student’s *t* test was used for the analysis with a *p* <0.05 being considered statistically significant

**Table 3:** Anthropometric, clinical and biochemistry characteristics of the male population

Variable	Men (*n* = 110)		*p*
	2007	2013	
Age (years)	17.53 (1.04)	23.44 (1.16)	<0.05
Wight (kg)	70.53 (13.20)	76.31 (12.49)	<0.05
BMI (kg/m2)	23.50 (4.21)	25.10 (3.73)	<0.05
Waist (cm)	81.12 (10.42)	86.06 (9.01)	<0.05
Systolic BP (mm Hg)	116.63 (11.65)	128.08 (12.83)	<0.05
Diastolic BP (mm Hg)	73.36 (8.57)	76.33 (10.21)	<0.05
Glucose (mg/dL)	87.46 (24.02)	86.21 (12.53)	≥0.05
Triglycerides (mg/dL)	160.58 (88.26)	92.64 (54.27)	<0.05
HDL Cholesterol (mg/dL)	41.79 (12.51)	37.42 (11.01)	<0.05
LDL Cholesterol (mg/dL)	107.17 (52.47)	96.87 (32.46)	≥0.05
Total Cholesterol (mg/dL)	179.31 (55.23)	152.56 (32.08)	<0.05

Data are shown as mean ± standard deviation

*BMI* body mass index; *BP* blood pressure; *LDL* low-density lipoprotein cholesterol; *HDL* high-density lipoprotein cholesterol. Student’s *t* test was used for the analysis with a *p* <0.05 being considered statistically significant

The mean BMI at baseline was 22.98 kg/m^2^, while at the end it was 24.37 kg/m^2^ (*p* < 0.05), with the male population showing a BMI in the range of overweight (25.10 kg/m^2^). The number of students with overweight and obesity increased from 24.9 to 37.1 % (*p* < 0.05). However, this increase was only statistically significant among men, the group in which mean weight gain after 6 years was about 6 kg.

The diagnosis of central obesity increased from 17.8 to 28.6 % (*p* < 0.05), and by gender it was only significant among women (12.6 vs 24.3 %). The components of metabolic syndrome in 2007 in order of importance were low HDL cholesterol (59.1 %), hypertriglyceridemia (38.1 %), hyperglycemia (25.8 %), and hypertension (13.6 %), while in 2013 HDL cholesterol remained as the most important factor (67.1 %), followed by hypertension (33.8 %), abdominal obesity (28.6 %), hyperglycemia (10.3 %) and hypertriglyceridemia (5.6 %). It is important to note that at the start of the study, a higher prevalence of low HDL cholesterol in women (66.9 51.8 %) was identified, which denoted statistical significance (*p* < 0.05). Hypertension prevalence increased significantly only in the male population. Both hypertriglyceridemia and hyperglycemia showed a decreasing trend with statistical significance (*p* < 0.05) throughout the study.

Based on the definition of the IDF, in 2007, metabolic syndrome was diagnosed in 9.8 % of the population, while in 2013, it was in 14.5 %. This did not represent a significant change (*p* ≥ 0.05) in the general population neither by gender.

Our study has important findings: most participants had at least one component of the metabolic syndrome (69.2 % in 2007 and 76.4 % in 2013); low HDL cholesterol is the most prevalent risk factor; and the male population had more marked metabolic abnormalities.

## Conclusions

The change in the prevalence of metabolic syndrome among our student population was not statistically significant. Low HDL cholesterol and hypertriglyceridemia were the main risks factors in the population less than 20 years of age, while in the population of 20–30 years, low HDL cholesterol and high blood pressure were the main risk factors (low HDL cholesterol affected two thirds of the population). Therefore, it is important the study of young populations in order to make a timely intervention in individuals at risk, given the high prevalence of metabolic abnormalities at an early age.

## Abbreviations

BMI: Body mass index
IDF: International Diabetes Federation
UANL: Universidad Autónoma de Nuevo León
HDL: High density lipoprotein cholesterol
LDL: Low density lipoprotein cholesterol
